# Psychometric properties of the emotional processing scale in individuals with psychiatric symptoms and the development of a brief 15-item version

**DOI:** 10.1038/s41598-022-14712-x

**Published:** 2022-06-21

**Authors:** Daniel Maroti, Erland Axelsson, Brjánn Ljótsson, Gerhard Andersson, Mark A. Lumley, Robert Johansson

**Affiliations:** 1 Department of Clinical Neuroscience, Karolinska Institutet, 171 65 Stockholm, Sweden; 2 Division of Family Medicine and Primary Care, Department of Neurobiology, Care Sciences and Society, Karolinska Institutet, Huddinge, Sweden; 3 Liljeholmen Primary Health Care Centre, Region Stockholm, Stockholm, Sweden; 4 Academic Primary Health Care Centre, Region Stockholm, Stockholm, Sweden; 5 Department of Behavioral Sciences and Learning, Department of Biomedical and Clinical Sciences, Linköping University, Linköping, Sweden; 6 Department of Psychology, Wayne State University, Detroit, MI USA; 7 Department of Psychology, Stockholm University, Stockholm, Sweden

**Keywords:** Psychology, Human behaviour

## Abstract

The 25-item Emotional Processing Scale (EPS) can be used with clinical populations, but there is little research on its psychometric properties (factor structure, test–retest reliability, and validity) in individuals with psychiatric symptoms. We administered the EPS-25 to a large sample of people (N = 512) with elevated psychiatric symptoms. We used confirmatory factor analysis to evaluate three a priori models from previous research and then evaluated discriminant and convergent validity against measures of alexithymia (Toronto Alexithymia Scale-20), depressive symptoms (Patient Health Questionaire-9), and anxiety symptoms (Generalized Anxiety Disorder-7). None of the a priori models achieved acceptable fit, and subsequent exploratory factor analysis did not yield a clear factor solution for the 25 items. A 5-factor model did, however, achieve acceptable fit when we retained only 15 items, and this solution was replicated in a validation sample. Convergent and discriminant validity for this revised version, the EPS-15, was r = − 0.19 to 0.46 vs. TAS-20, r = 0.07− 0.25 vs. PHQ-9, and r = 0.29− 0.57 vs. GAD-7. Test–retest reliability was acceptable (ICC = 0.73). This study strengthens the case for the reliability and validity of the 5-factor structure of the EPS but suggest that only 15 items should be retained. Future studies should further examine the reliability and validity of the EPS-15.

## Introduction

According to the emotional processing model (EPM), acknowledging emotions and finding adaptive ways of expressing them are foundational to adaptive coping with stressful life events^1–3^. The EPM proposes that disruptions in coping can occur when people avoid or suppress their emotions, which will inhibit emotional processing and give rise to difficulties with unprocessed or uncontrolled emotions, which in turn may contribute to the development of psychiatric and somatic symptoms.

The concepts of emotional processing and the EPM are closely related to the concepts of emotional regulation and the process model of emotion regulation^1,4^. Both models, for example, recognize situational avoidance as a strategy to cope with upsetting events. An important distinction, however, is that “emotional regulation” refers primarily to attempts at influencing emotions to reach adaptive goals (such as stable mental health), whereas “emotional processing” refers to what disrupts the overarching process of overcoming difficult or stressful life events more broadly^1,4,5^. This difference in focus—strategies to manage emotions versus obstacles to natural emotional processing—leads to the study of different phenomena. According to the EPM, for example, it is important to determine whether people are experiencing difficulties understanding emotions, because such a lack of understanding is likely to hinder emotions being processed. In contrast, the process model of emotional regulation does not explicitly address this, as a lack of understanding emotions is not a strategy people can employ in order to regulate emotions.

The Emotional Processing Scale (EPS) was developed to enable the study of emotional processing in accordance with the EPM. The original version of this scale comprised 38 items and 8 subfactors^1^ which was later shortened to a 25-item version (EPS-25) which has five subscales purportedly corresponding to five facets of emotional processing^2^: *Avoidance* refers to strategies to avoid triggering emotions surrounding an event or situation. *Suppression* refers to attempts to not show feelings outwardly. *Impoverished emotional experience* captures aspects of “alexithymia”; that is, difficulties identifying own emotions related to an event. The final two subscales assess consequences of inadequate emotional processing: *Signs of unprocessed emotions* can, for example, manifest as nightmares, whereas *Unregulated emotions* can be expressed in temper tantrums.

### Emotional processing deficits in individuals with elevated psychiatric symptoms

According to the EPM, a lack of adequate emotional processing will be associated with psychiatric and medically unexplained somatic symptoms^1,2,5^. Consistent with this hypothesis, emotional processing deficits have been identified in patients with psychogenic nonepileptic seizures^6^, functional neurological symptoms^7^, irritable bowel syndrome (IBS)^8^, chronic (back) pain^9,10^, post-traumatic stress disorder (PTSD)^11,12^, substance use disorder^12^, bipolar disorder^10^, and anxiety disorders^13^. Emotional processing deficits do not appear to be specific to psychiatric populations, however, as deficits have also been identified in populations of people with medical conditions such as ischemic heart disease^14^, multiple sclerosis^10,15^ and type 2 diabetes^16^.

Moreover, although several psychometric studies of the EPS-25 have been published, most of these studies have examined the EPS-25 in non-psychiatric samples, such as healthy participants, medical patients, or a combination of these^17–20^. Of the few validation studies on people with psychiatric problems, one had a small highly selected sample (24 patients with bipolar disorder hospitalized for depression)^10^, and the other investigated an unspecified population (people referred to a psychologist for various mental health problems)^2^. Thus, further validation of the factor structure of the EPS-25 in a large sample of people with psychiatric symptoms is needed as an initial step in determining the validity of this measure as a predictor of the development and maintenance of both psychiatric disorders and functional somatic syndromes.

### Internal consistency and dimensionality

There have been several psychometric studies on the EPS-25, and studies generally report excellent internal consistency for the whole scale, and fair to good internal consistencies for the subscales^2,10,17,19–23^. In Baker et al.’s (2010) original article, the EPS-25 was administered to a mixed sample of 690 medical patients, psychiatric patients, and healthy controls^2^. Exploratory factor analysis revealed the 5-factor structure described above. Moreover, Gay et al. (2019) administered the EPS-25 to a combined sample of 1176 medical patients, hospitalised patients with bipolar disorder, city hall employees, and students^10^. An exploratory factor analysis with five factors defined a priori revealed factor loadings similar to those reported by Baker et al. (2010), although five items had cross-loadings over 0.30.

Other attempts to replicate the original 5-factor solution, however, have failed. Using two community samples (N = 1172), Spaapen (2015) conducted a confirmatory factor analysis of the 5-factor solution suggested by Baker et al. (2010), but this factor structure did not achieve acceptable fit, either in its original form or after dropping the three most problematic items^18^. Orbegozo et al. (2018) investigated the factor structure of the EPS-25 with confirmatory factor analysis in school and university students (N = 605), but the original 5-factor model did achieve an acceptable fit^20^. Moreover, neither Kharamin et al. (2021), who used a confirmatory factor analysis of the EPS-25 among university students (N = 1283)^19^, nor Lauriola et al.^17^, who administered the EPS-25 to a combined sample of gastrointestinal patients and healthy participants (N = 696), replicated the original 5-factor structure.

Given that replication of the original findings of Baker et al. (2010) has proven difficult, several authors have explored alternative structures. Spaapen (2015) investigated a 2-factor model, with *Suppression* as one factor, and the other four subscales representing another factor, but confirmatory factor analysis did not establish a convincing model fit^18^. Other authors have added a second-order latent “emotional processing” factor (i.e., a general emotional processing capacity factor) to the five subfactors, which increased model fit^17,19^. Another solution has been to reduce the number of items or move items among factor to achieve adequate fit^20^.

Taken together, despite difficulties in replicating Baker et al.’s (2010) original findings, most previous studies support a 5-factor model, either with a second-order latent “emotional processing” factor^17,19,20^ or without one^2,10^, although establishing a 5-factor model required revisions in some studies, such as reducing items^18,20^. Thus, the structure of the EPS-25 proposed by Baker et al. (2010) is far from consistently replicated and needs further study.

### Convergent and discriminant validity

The validity of a measure can be assessed by the correlations it has with other measures of relevance. Because the EPS-25 purports to assess dysfunctional emotional processing, it should correlate relatively highly (convergent validity) with measures of similar constructs, such as alexithymia (i.e., difficulties identifying and expression emotions), but correlate less strongly (discriminant validity) with measures of constructs not directly part of emotional processing (such as depression).

The convergent validity of the EPS has been studied in relation to concepts such as emotional control^1^, emotional regulation^10,20^, and alexithymia^1,2^. In particular, the *Impoverished emotional experience* factor of the EPS-25 has been proposed^1,2^ as similar to the alexithymia facet of difficulties identifying one’s own feelings (measured by the Toronto Alexithymia Scale-20); however, surprisingly low concurrent validity (*r* = 0.35) has been found^10,24^. In addition, the discriminant validity of the EPS-25 has been mixed, particularly findings of a larger than hypothesized relationship between the scale and measures negative affect such as anxiety (r = 0.47 to 0.59) and depressive symptoms (r = 0.48 to 0.63)^10,19,20^. Emotional processing is also proposed to have discriminant validity with another facet of the alexithymia^1^, *external oriented thinking* (Toronto Alexithymia Scale-20, facet 3), and studies have found that the EPS-25-total is uncorrelated with this subscale^10^.

### Test–retest reliability

Only two studies have investigated EPS-25 test–retest reliability. In a convenience sample of 17 healthy people, test–retest reliability over 4 to 6 weeks for the entire scale was 0.74^2^. In another study, the 4-week test–retest correlation was 0.91 among 80 students^19^. Clearly there is need for further studies of test–retest reliability of the EPS-25, particularly in populations with elevated psychiatric symptoms.

### Study aims and hypotheses

The *planned aim* of this study was to conduct a structural validation of the EPS-25 in a sample of patients with elevated psychiatric symptoms. We hypothesized that the EPS-25 would show either a 5-factorial structure (with or without a higher-order, general emotional processing capacity latent factor) consistent with the description of Baker et al. (2010), or a 2-factorial structure consistent with Spaapen’s (2015) description of the two factors. We also hypothesized that the internal consistency would be good (α ≥ 0.80) for the EPS-25 total scale and at least fair for the five subscales (α ≥ 0.60). We also tested convergent validity of the EPS-25 with the Toronto-Alexithymia Scale-20 (TAS-20), hypothesizing a relatively high correlation (r = 0.50 to 0.75) with the TAS-20 factor 1 (Difficulty Identifying Feelings). For discriminant validity, we hypothesized that the association of EPS-25 with anxiety and depression would be lower (r = 0.25 to 0.50) than the EPS relationship with TAS-20. We also expected that the EPS would not be correlated with external oriented thinking from the TAS-20. A last aim of this study was to evaluate EPS test–retest reliability, which was hypothesized to have an adequate test–retest reliability (i.e.**,** ICC ≥ 0.60) over approximately 1 week. Finally, as we conducted analyses of the EPS-25, we developed an additional aim, which was to investigate whether a shorter version of the scale might be psychometrically sound.

## Method

### Sources of the data and participants

Data for this study were taken from the baseline (pre-intervention) assessment of four clinical trials of internet-delivered psychodynamic treatment^25–28^. These trials were conducted on people with an anxiety disorder or depression^25^, social anxiety disorder^26^, or somatic symptom disorder^27^. In all studies, adult participants were recruited from the community by advertisement and were enrolled using a safe internet platform. The main common exclusion criterion were the presence of other major psychiatric conditions, where outpatient care would be more appropriate (e.g., psychosis, suicidal ideation). In add trials, participants completed self-report questionnaires, from home, on a secure web platform. Traffic with the web platform was encrypted, and all studies proceeded in accordance with relevant data management and privacy legislation. For further information about recruitment procedures and patient criteria, see the original studies. The research was performed in accordance with the Declaration of Helsinki, all participants (N = 512) provided informed consent, and all four trials were conducted in accordance with relevant regulations and approved by the appropriate regulatory authorities (Regional Ethics Board of Linköping: 2011/400–31, 2013/361–31; Swedish Ethical Review Authority: 2019–03,317, 2020–03,490). ClinicalTrials.gov identifiers are: NCT01532219, NCT02105259, NCT04122846 and NCT04751825. Data are available on request by the corresponding author.

For factor analyses of the EPS-25, data from all four trials were used. For analyses of discriminant and convergent validity, data were taken from the baseline of only one trial^28^ that had data on the other validations measures. To calculate test–retest reliability, data came from only one trial^27^ that had an adequate number of days (Range: 5–12 days) between the first and second administrations of the EPS-25 prior to treatment.

### Measures

*The Emotional Processing Scale* (EPS-25) has 25 items which are rated on a 10-point scale from 0 (*completely disagree*) to 9 (*completely agree*). The mean of all items yields the overall score, and means of the five, 5-item subscales are also calculated: *avoidance*, *suppression*, *impoverished emotional experience*, *signs of unprocessed emotions,* and *unregulated emotions*. The EPS-25 was translated from English to Swedish by three people fluent in both languages, by using multiple back-and-forth rounds until a satisfactory translation was reached^29^. (For further details, see^30^).

*The Toronto Alexithymia Scale* (TAS-20) has 20 items rated on a scale of 1 (*strongly disagree*) to 5 (*strongly agree*)^31,32^, and fields a total score (range: 20 to 100) as well as scores on three facets or subscales: *difficulty identifying feelings* (DIF), *difficulty describing feelings* (DDF), and *externally-oriented thinking* (EOT). The TAS-20 has shown both good internal consistency and test–retest reliability in the Swedish population^33^.

*The Patient Health Questionnaire-*9 (PHQ-9) assessed depressive symptom severity. The nine items rated 0 to 3 and summed (range: 0 to 27). The PHQ-9 has good psychometric properties, including an internal consistency in the range of Cronbach’s α = 0.86–0.89^34^.

*The Generalized Anxiety Disorder-*7 scale (GAD-7; Spitzer et al., 2006) assesses anxiety symptom severity. The seven items are rated 0 to 3 and summed (range: 0 to 21). Internal consistency is excellent (Cronbach’s α = 0.92)^34^.

### Statistical analyses

Within a confirmatory factor analytic (CFA) framework, we used all available EPS-25 data (N = 512) and tested three different possible factor solutions in R 4.1.0 (R Core Team, 2016) with lavaan 0.6–8: (1) a 5-factor model corresponding to the original solution presented by Baker et al.^2^; (2) a 5-factor model with a second order “emotional processing” latent variable as found to be adequate in previous studies^17,19^; and (3) a 2-factor model with *suppression* and *other* factors as discussed by Spaapen (2015)^18^. Criteria for good model fit were: CFI and TLI at least 0.90 (ideally 0.95), RMSEA and SRMR < 0.08, and lowest possible AIC and BIC^36^.

Using Jamovi^37^, we analysed EPS internal consistency (Cronbach’s α), investigated convergent and discriminant validity using Pearson correlations, and estimated test–retest reliability using the intraclass correlation coefficient (ICC). For the *α* statistic, values ≥ 0.90 are commonly regarded excellent, ≥ 0.80 good, and ≥ 0.70 acceptable. Importantly however, α also decreases substantially with fewer items in a scale; for example, going from 5 to 3 items could be expected to lower α around 0.10–0.15 units. For the *r* statistic, values around 0.50 are commonly regarded as indicative of a strong/large association, 0.30 is moderate/medium, and 0.10 is weak/small^38^. For the ICC, values ≥ 0.75 are commonly regarded excellent, ≥ 0.60 good, ≥ 0.40 fair, and < 0.40 poor^39^.

### Ethical approvals

The research was performed in accordance with the Declaration of Helsinki, all participants (N = 512) provided informed consent and all four clinical trials were conducted in accordance with relevant regulations and approved by the appropriate regulatory authorities: (Regional Ethics Board of Linköping: 2011/400-31, 2013/361-31; Swedish Ethical Review Authority: 2019–03,317, 2020–03,490). ClinicalTrials.gov identifiers are: NCT01532219, NCT02105259, NCT04122846 and NCT04751825.

## Results

### Psychometric analysis of the EPS-25

#### Confirmatory factor analysis (CFA) of EPS-25

Because kurtosis was high for several items, and some items had many zero scores, models were fit using maximum likelihood estimation with robust (Huber-White) standard errors and a scaled test statistic. In the CFA using all data, none of the three a priori models of EPS-25 achieved adequate fit (see Table 1). Theoretically sound changes in accordance with modification indices, including the removal items (6, 8, 14, 17, 23) and the specification of reasonable residual covariance (for example 18, 19), also did not yield acceptable model fit.

**Table 1:** Fit indices and other key dimensionality parameters derived from factor analysis of the Emotional Processing Scale-25.

Framework	Model	Data	Items	χ^2^	χ^2^/df	P	CFI	TLI	RMSEA (90% CI)	SRMR	AIC	BIC	Cross-loadings or as indicated	All loadings < 0.4	Factor corr
**Phase 1: test of a priori models**															
CFA	A priori 5F two-tier	Total	25	1060	3.9	< 0.001	0.84	0.82	0.082 (0.077, 0.087)	0.079	57,211.8	57,443.6	MOI: 6, 14, 17, 19, 23	4, 14	0.66–0.96^a^
CFA	A priori 5F	Total	25	1016	3.8	< 0.001	0.85	0.83	0.080 (0.075, 0.085)	0.074	57,166.8	57,419.6	MOI: 6, 14, 17, 18, 19, 23	4, 14	0.42–0.84
CFA	A priori 2F	Total	25	1323	4.8	< 0.001	0.78	0.76	0.094 (0.089, 0.099)	0.085	57,516.0	57,730.9	MOI: 5, 6, 14	4, 14	0.63
**Phase 2: exploratory modeling**															
EFA	1F	Training	25/23^c^	1078	3.9	< .001		0.67	0.107 (0.100, 0.114)			− 447	Not applicable	4, 14	Not applicable
EFA	Free 2F^b^	Training	25/21^c^	667	2.7	< .001		0.81	0.080 (0.073, 0.088)			− 725	≥ 0.4: 14	4, 5, 6, 12	0.66
EFA	Free 5F	Training	25/22^c^	300	1.6	< 0.001		0.93	0.049 (0.039, 0.060)			− 725	≥ 0.4: 6, 8, 19, 23	9, 10, 14	0.14–0.74
CFA	EPS-15 5F^d^	Training	15	125	1.6	0.001	0.96	0.95	0.049 (0.031, 0.065)	0.046	17,875.0	18,016.8	MOI: 2, 3, 5, 8, 9, 21, 22, 24, 25	None	0.45–0.75
**Phase 3: validation**															
CFA	EPS-15 5F^d^	Validation	15	180	2.2	< 0.001	0.92	0.89	0.075 (0.060, 0.090)	0.053	16,987.9	17,127.7	MOI: 5, 16	None	0.36–0.83

Robust fit indices from confirmatory factor analysis. Note that because these models are fitted on different data (the total, training, and validation sample) all values are not directly comparable. Note also that the 2 and 5 factor solutions derived from exploratory factor analysis where all items are allowed to freely load on all factors (that is, cross-loadings are estimated freely over all factors) do not necessarily correspond to other published factor solutions such as those of Baker et al. (2010) or Lauriola et al. (2021). Due to software limitations, fewer fit indices are provided for the EFA models.

*2F* two-factor, *5F* five-factor, *AIC* Akaike information criterion, *BIC* Bayesian information criterion, *CFI* comparative fit index, *EPS-15* 15-item version of the emotional processing scale, *MOI* 20 largest modification indices (1-df), *RMSEA* root mean square error of approximation, *SRMR* standardized root mean square residual, *TLI* Tucker Lewis index.

^a^Loadings on the latent “emotional processing” factor.

**Figure 1:**
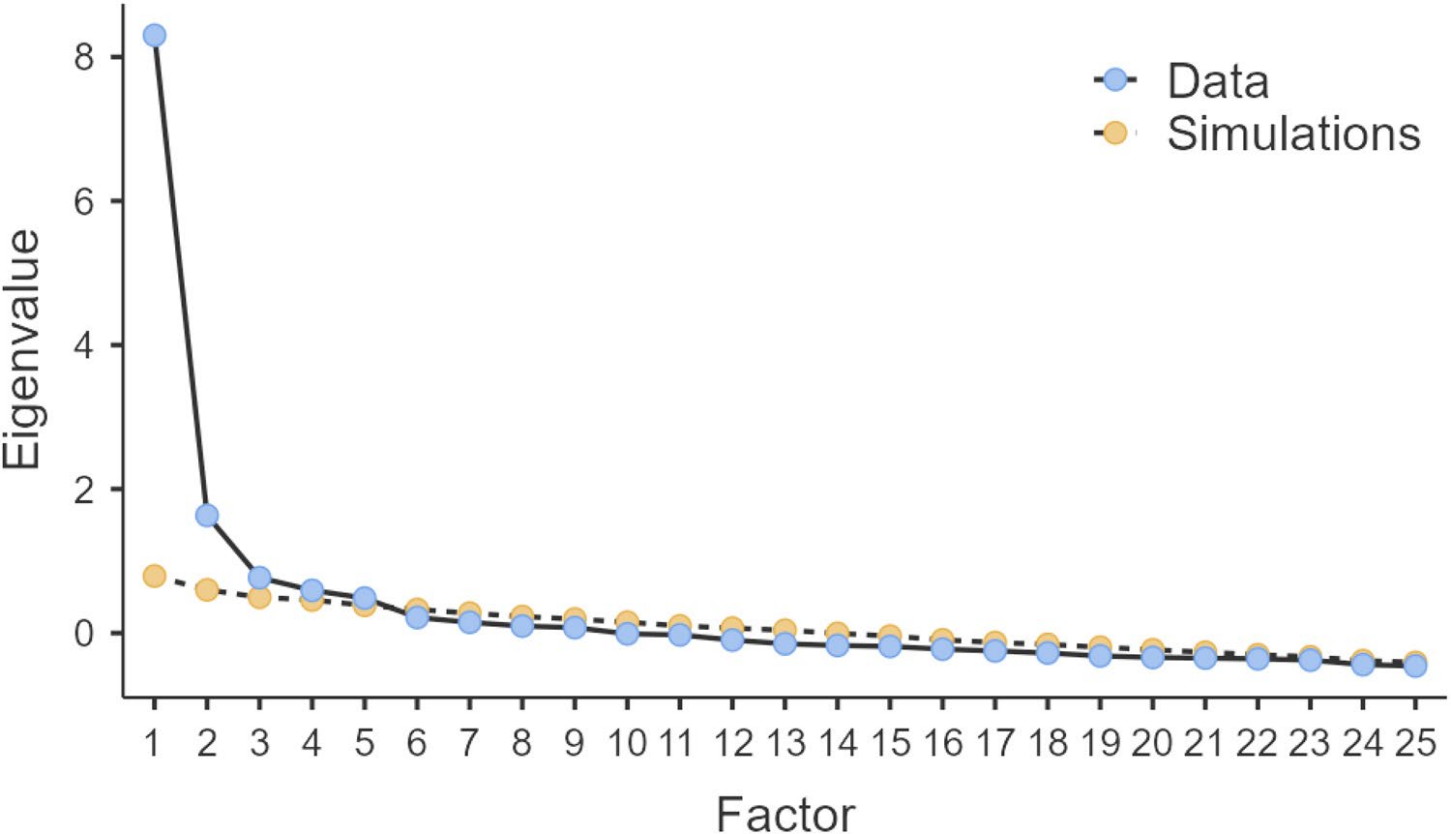
Scree plot with reference eigenvalues based on parallel analysis (“Simulations”).

^b^Arguably the most promising model according to the scree plot, with a clear increase in eigenvalue and deviation from factors derived from simulated data occurring between factor 3 and 2 (see Fig. 1).

^c^Note that while all 25 items were included in the analysis, none of the factor solutions derived from EFA resulted in all 25 items having factor loadings of at least 0.4 on at least one factor. For example, in the 5-factor solution derived from EFA, items 9, 10, and 14 did not load 0.4 or higher on any factor, which means that this was in effect a 22-item solution.

^d^This is the final 15-item factor solution, reached primarily via stepwise modification of the original CFA a priori 5F (non-two-tier) model. See the main text for details.

#### Internal consistency of EPS-25

Cronbach’s alpha was excellent for the EPS-25 overall score (*α* = 0.92), good for the *avoidance* (*α* = 0.87) and *impoverished emotional experience* subscales (*α* = 0.83), and acceptable (*α* = 0.67 to 0.75) for the other three subscales.

#### Convergent and discriminant validity of EPS-25

As seen in Table 2, the EPS-25-total showed strong correlations with anxiety (*r* = 0.66) and depression (*r* = 0.59). A moderate correlation of i*mpoverished emotional experience* and the alexithymia factor *difficulty identifying feelings* (TAS-20, factor 1) was found (*r* = 0.35) but also a weak correlation (*r* = 0.21) with *externally-oriented thinking* (TAS-20, factor 3).

**Table 2:** Correlations between the Emotion Processing Scale (EPS-25) and measures of alexithymia (TAS-20), anxiety (GAD-7), and depression (PHQ-9) (N = 74)^28^.

	EPS-25 total	Avoidance	Suppression	Impoverished emotional experience	Signs of unprocessed emotions	Unregulated emotions
**TAS-20**						
Total	0.57***	0.57***	0.29***	0.35***	0.46***	0.61***
Describing feelings	0.49***	0.53***	0.20**	0.27***	0.40***	0.56***
Identifying feelings	0.65***	0.52***	0.45***	0.48***	0.53***	0.59***
Externally oriented thinking	0.21**	0.33***	0.02	0.04	0.15*	0.31***
GAD-7	0.66***	0.40***	0.57***	0.61***	0.54***	0.50***
PHQ-9	0.59***	0.43***	0.44***	0.48***	0.47***	0.49***

*P < 0.05, **P < 0.01, ***P < 0.001.

#### Test–retest reliability of the EPS-25

Test–retest reliability of the EPS-25 was conducted on 51 participants from Maroti et al.^27^ over an approximately 1-week period (M = 8.06, SD = 1.35, range: 5–12 days). The test–retest reliability was excellent (ICC = 0.76).

### Development and validation of the EPS-15

Because we could not replicate any of the a priori factor structures for the 25-item version of EPS using confirmatory factor analysis, we attempted to find a more suitable factor solution using exploratory factor analysis. Also, although the EPS-25 showed good internal consistency and test–retest reliability, discriminatory validity was unsatisfactory. The EPS-25 had large relationships with depression and anxiety and also was correlated with externally-oriented thinking of the alexithymia construct—findings that are not predicted by Baker et al.^1^.

To find and validate a better factor solution, the sample was split into training (n = 262) and validation (n = 250) subsamples by randomization. We deemed these sample sizes adequate for factor analysis considering that they were close to the common recommendation of 300^40^, and we expected communalities to be at least moderate^41^. We then conducted an exploratory factor analysis (EFA) based on principal axis factoring with promax (oblique) rotation, with the intention of finding an empirically- and theoretically-sound factor solution for the data. In these analyses, we explored 1-, 2-, and 5-factor solutions as informed by the scree plot (see Fig. 1) and our theoretical understanding of the scale and the emotional processing model. We wanted to achieve distinct factors as characterized by factor loadings of all items ≥ 0.30 (ideally ≥ 0.40), with few or no substantial cross-loadings, and at least three items loading on each factor.

#### Exploratory factor analysis (EFA) of the EPS-15

The training data set was suitable for factor analysis (Barlett’s test *p* < 0.001; KMO = 0.91). The screen plot (Fig. 1) was inconclusive; the knee being indicative of 1—or more probably 2—factors, while parallel analysis resulted in weaker eigenvalues up to factor 5, even though the difference was small for factors 3 to 5. However, none of the freely estimated 1-, 2-, or 5-factor solutions for the EPS-25 could achieve acceptable fit with distinct factors, meaning that each of all 25 items had factor loadings of at least 0.40 with minimal cross-factor loadings (see Table 1).

#### Development and dimensionality of the EPS-15

As neither confirmatory nor exploratory factor analysis resulted in an acceptable factor solution for the EPS-25, we sought to develop a shorter scale scale, the EPS-15, with a more distinct factor structure. We intended to identify a subset of the EPS-25 items that would allow for stronger model fit and distinct, yet correlated, factors. To achieve this goal, we based the item selection process on the best fitting 25-item CFA model (this had 5 factors corresponding to the conventional subscale scoring), and the stepwise deletion of items, and addition of covariance if theoretically feasible, based on modification indices and our theoretical understanding (see Table 1), until exactly 3 items remained for each of the 5 factors. We subsequently validated this model in the validation data set.

#### Further statistical and theoretical considerations

Based on modification indices for the 25-item CFA training data 5-factor solution, item 6 (“Could not express feelings”) was moved to the *impoverished emotion* factor. The following 10 items were then removed step by step: 23, 17, 8, 14, 5, 6, 20, 19, 11, and 12. We found the correlation between the *unregulated emotions* and *suppression* factors to be unsatisfactory (r = 0.31, i.e., clearly lower than 0.40) and therefore replaced item 18 (“Felt urge to smash something”) with item 8 (“Reacted too much to what people said or did”) to increase the correlation between subscales to an acceptable *r* = 0.45. The resulting five-factor model on 15 items (three per factor) achieved acceptable model fit in terms of the RMSEA (0.050, [90% CI 0.033, 0.066]), SRMR (0.046), CFI (0.96), and TLI (0.95).

To reach the best suiting 15 items of the 25 items available, the items were also scrutinized for adequate content validity by D.M and R.J, who, at that time, did not know of what 15 item the structural validation had suggested (see Table 3). The items were deemed either fully indicative of its subfactor (coded as “yes”), only partly so (coded as “borderline”) or not an adequate description of content validity (coded as “no”). Following these considerations, one additional change of the EPS-15 was made as we did not regard item 10 (“My feelings did not seem to belong to me”) as a convincing example of *impoverished emotional experience* understood as alexithymia^42^ and therefore instead reintroduced item 5 (“My emotions felt blunt/dull”; see Table 3).

**Table 3:** Comparison between blinded theoretical judgment (by DM and RJ), initial empirical suggestion of problematic items, and the final EPS-15.

			Theoretical judgment	Sum of EPS-25 modification indices indicative of potential cross-loadings^a^	EPS-15 final item selection	Factor loadings in validation sample
Subscale	#	Items paraphrased				
Suppression	1	Smothered feelings	Yes	0	X	0.78
	6	Could not express feelings	No	145		
	11	Kept quiet about feelings	Borderline	13		
	16	Bottled up emotions	Yes	0	X	0.85
	21	Tried not to show feelings	Yes	0	X	0.76
Signs of unprocessed Emotions	2	Unwanted feelings kept intruding	Yes	0	X	0.77
	7	Emotional reactions lasted more than a day	Borderline/Yes	0	X	0.71
	12	Repeatedly experienced the same emotion	Borderline/Yes	13		
	17	Overwhelmed by emotions	Borderline	73		
	22	Thinking about same emotion again and again	Yes	0	X	0.74
Unregulated emotions	3	When upset difficult to control what I said	Yes	0	X	0.68
	8	Reacted too much to what people said or did	Yes	11	X	0.79
	13	Wanted to get own back on someone	Yes	0	X	0.51
	18	Felt urge to smash something	Yes	22		
	23	Hard to wind down	Borderline	78		
Avoidance	4	Tried to avoid things that might make me upset	Yes	0	X	0.40
	9	Talking about negative feelings made them worse	Borderline	0	X	0.67
	14	Tried to talk only about pleasant things	Borderline/Yes	50		
	19	Could not tolerate unpleasant feelings	No	12		
	24	Avoided looking at unpleasant things	Borderline/Yes	0	X	0.69
Impoverished emotional experience (“alexithymia”)	5	Emotions felt blunt/dull	Borderline	11	X	0.47
	10	Feelings did not seem to belong to me	No	0		
	15	Hard to work out if I felt ill or emotional	Yes	0	X	0.61
	20	Seemed to be a big blank in feelings	Borderline	0		
	25	Strong feelings but not sure if emotions	Borderline/Yes	0	X	0.73

EPS-15 Emotional processing Scale. Yes: endorsed as an item typical what the subscale is supposed to measure. No: not endorsed. Borderline: neither typical nor atypical.

^a^This is the sum of all modification indices pertaining to item-factor cross-loadings for each item, as based on the top 30 modification indices for the a priori 5-factor solution for the EPS-25 when fitted on the training data. Note that while there is considerable overlap between blinded theoretical judgments, these modification indices, and the final EPS-15, the reduction of the number of items from 25 to 15 was an iterative process where modification indices were examined for several intermediate scale forms, which for example is why we ultimately decided to include item 5 over items 10 and 20 in the EPS-15.

The resulting 15-item 5-factor model (from both statistical and theoretical considerations) had improved model fit in the training data and acceptable, though not ideal, fit in the validation data (see Table 1). The average variance extracted was 47% in both the training and validation data. All factor correlations and factor loadings were 0.40 or higher in the training data and remained so in the validation data, except for the correlation between the *unregulated emotions* and *suppression* factors, which dropped to 0.36 in the validation.

#### Internal consistency of the EPS-15

As shown in Table 4, Cronbach’s alpha was good for the EPS-15 overall scale (*α* = 0.87) and for the *suppression* subscale (*α* = 0.83), and acceptable (*α* = 0.62–0.76) for the remaining subscales. The composite reliability coefficient (ω) was almost identical.

**Table 4:** Internal consistency for the Swedish validation EPS-25 and EPS-15.

	Cronbach’s alpha	
	EPS-25	EPS-15
EPS total	0.92	0.87
Avoidance	0.87	0.62
Suppression	0.75	0.83
Impoverished emotional experience	0.83	0.64
Signs of unprocessed emotions	0.67	0.76
Unregulated emotions	0.79	0.69

#### Convergent and discriminant validity of EPS-15

As seen in Table 5, the EPS-15 had a strong correlation with anxiety (*r* = 0.57). but a weak correlation with depressive symptoms (*r* = 0.25). Moreover, we found a moderate correlation between *impoverished emotional experience* and the alexithymia factor *difficulty identifying feelings* (*r* = 0.46), but no significant correlation with *external oriented thinking style* (TAS-20, factor 3).

**Table 5:** Correlations between the 15-item Emotional Processing Scale (and subscales) with Alexithymia (TAS-20), anxiety (GAD-7), and depression (PHQ-9 (N = 74)^28^.

	EPS-15 total	Avoidance	Suppression	Impoverished emotional experience	Signs of unprocessed emotions	Unregulated emotions
**TAS-20**						
Total	0.34**	0.24*	0.39***	0.41***	0.13	0.13
Describing feelings	0.30*	0.23	0.45***	0.30**	0.09	0.05
Identifying feelings	0.47***	0.31**	0.35 **	0.46***	0.33**	0.30**
Externally oriented thinking	− 0.04	− 0.03	0.09	0.13	− 0.19	− 0.11
GAD-7	0.57***	0.50***	0.29*	0.34**	0.49***	0.47***
PHQ-9	0.25*	0.27*	0.32**	0.19	0.08	0.07

*P < 0.05, **P < 0.01, ***P < 0.001.

#### Test–retest reliability of the EPS-15

The test–retest reliability for the EPS-15 over approximately 1 week (M = 8.06 days, SD = 1.35, range: 5–12 days) was good (ICC = 0.73)*.*

## Discussion

Based on data from 512 individuals with elevated psychiatric symptoms, we could not find a satisfactory factor solution for the 25-item Emotional Processing Scale. This led us to develop a briefer 15-item version of the scale, the EPS-15, for which we found an acceptable 5-factor solution that we validated using a split sample strategy. EPS-15 had good internal consistency and test–retest reliability and demonstrated discriminant validity from the construct of depressive symptoms, although less so from anxiety symptoms.

### Inconsistent findings pertaining to the EPS-25

Evidence pertaining to the factor structure of the EPS-25 was inconsistent. On the one hand, the training data scree plot appeared to be indicative of one or probably two factors (suppression vs. other). On the other hand, in parallel analysis, higher eigenvalues were obtained up to the fifth factor, and a 5-factor solutions was most promising in terms of model fit under both CFA and EFA. Most previous studies appear to speak for some type of 5-factor model, sometimes with a second order latent “emotional processing” factor^17,19,20^, but sometimes not^2,10^. There are several potential explanations for the difficulties we encountered in replicating these 5-factor solutions. In CFA, as suggested by Lauriola et al.^17^, poor fit may have been a result of this type of model not allowing for cross-loadings over factors in the same manner as EFA, which was used in the original publication^2^. However, undue reliance on cross-factor loadings for model fit could also indicate poorly defined factors. The existence of weak main factor loadings, in combination with strong cross-loadings, speaks against scoring of the five conventional subscales, and the brief EPS-15 did indeed achieve acceptable fit under CFA. Importantly, in our data, the EPS-25 5-factor EFA model, for which item factor loadings were estimated freely, was also not satisfactory, especially as it had a pattern of factor loadings that was clearly inconsistent with the conventional scoring of the EPS-25 subscales; items 6, 8, 19, 23 exhibiting cross-loadings and items 9, 10, 14 not belonging to any factor.

### Properties and potential advantages of the EPS-15

The factor structure of the EPS-15 brief scale appeared to replicate over the testing and validation samples. Considering that all item factor loadings were 0.40 or higher, and acceptable model fit was achieved in the CFA framework without the need to specify cross-loadings (that is, each item loaded only on its intended factor), the factor solution appeared to support scoring of five separate subscales.

The EPS-15 demonstrated discriminant validity from the construct of depressive symptoms, although to a lesser degree of anxiety symptoms. In previous studies, the EPS-25 has not shown adequate discriminant validity from either depressive or anxiety symptoms^10,19,20^, and we also found that the EPS-25 had an substantial relationship with both anxiety symptoms and depressive symptoms. Moreover, it has been suggested^1^ that the EPS should not correlate with the Toronto Alexithymia Scale-20, externally-oriented thinking subscale, but in previous studies, subscales of the EPS-25 have been found to correlate significantly, albeit weakly, with this alexithymia facet^24^. Moreover, two of the EPS-25 subscales in this study had moderate correlation with externally-oriented thinking, but this was not the case for the EPS-15, further strengthening EPS-15 discriminant validity.

In the only two previous studies that have researched EPS-25 test–retest reliability, this has found to be good to excellent^2,19^. In the current study, both EPS-25 and EPS-15 demonstrated good test–retest reliability over approximately 1 week.

Compared to the EPS-25, the shorter EPS-15 will be easier to administer and complete, especially when used in combination with other scales (such as in routine care screening batteries), when space is limited (such as in epidemiological research), and when repeated measurements are conducted (such as during treatment). Moreover, without losing any of the important psychometric strengths of the EPS-25 (i.e., internal consistency, test–retest reliability), the EPS-15 was better able to discriminate emotional processing from depressive symptoms and facets of the alexithymia construct.

### Limitations

There are several limitations of this study. The test–retest reliability and the convergent and discriminant validation analyses was conducted on a small sample. Also, the use of randomization to form the testing and validation subsamples did not result in as stringent of a validation as a true replication in data from an entirely new sample.

Participants in the current analyses self-selected to take engage in internet-delivered emotion-focused treatments, and it is not clear how similar this population is to those in clinical practice or in the community, thus limiting generalizability of the findings. Moreover, the sample used for studying concurrent and discriminant validity were all diagnosed with somatic symptom disorder, and generalization to other samples, including healthy people and those with other psychiatric conditions, is limited.

The difficulties replicating the Baker et al. (2010) original 5-factor structure with adequate factor loadings for 25 items in this study could partly stem from our population studying individuals with elevated psychiatric symptoms. Almost all previous validation studies were on healthy samples and/or medical populations^17–20^. Emotional processing difficulties might differ between healthy people and those with elevated psychiatric symptoms. However, replicating the original findings of Baker et al. (2010) has been consistently difficult in other validation studies of healthy and/or medical samples as well^17–20^. Moreover, to find an adequate factor solution, previous authors have excluded problematic items—in effect, shortening the original 25-item scale^18,20^. We believe that our difficulty replicating Baker et al. (2010) stems not only from differences in population but differences in factor analytic procedures and “problematic” items with cross loadings initially used to develop the psychometric properties of the EPS-25.

### Overall discussion and future studies

Despite the interest in modelling and measuring emotional processing, considerable challenges remain, and many questions require further investigation. Further structural validation studies of the EPS-15 comparing different populations (i.e., comparing psychiatric to healthy populations) would be of interest. EPS-15 convergent validity should further be clarified, relating it to other measures of emotional processing, such as emotional awareness (measured by Level of Emotional Awareness Scale) or emotional regulation (measured by Difficulties of Emotional Regulation Scale). Further item analysis of the EPS-15 would be useful, given that the average variance extracted was around 50% (barely acceptable). It would also be preferable to further reduce the error variance of the EPS-15, for example by replacing or rephrasing items with relatively low factor loadings (e.g., EPS-25 equivalent items 4, 5, 13) or a particularly poor theoretical basis (e.g., EPS-25 equivalent items 5 and 9; see Table 2).

Despite continued challenges, we believe that this study contributes to the existing body of knowledge in several ways. First, an adequate test–retest reliability in a sample with elevated psychiatric symptoms (or more specifically somatic symptom disorder) has not been previously demonstrated. This is important as the EPS-25 is recommended for longitudinal psychotherapy research^1,10,17^. Second, despite reducing the number of items to 15, a 5-factor solution was retained, which is in line with previous research. This factor structure is important for clinicians, for example, who might desire to describe or address patient’s difficulties in emotional processing in five different domains. Third, we believe that this study overcomes some of the methodological shortcomings in previous research of the EPS-25, and the EPS-15 holds promise in populations with psychiatric symptoms. In conclusion, the EPS-15 is a promising short-form questionnaire for basic and clinical studies, although both further research on reliability and validity should be conducted.
